# Tricuspid valve obstruction and right heart failure due to a giant right atrial myxoma arising from the superior vena cava

**DOI:** 10.1186/1749-8090-8-200

**Published:** 2013-10-30

**Authors:** Zheng-hua Xiao, Jia Hu, Da Zhu, Ying-kang Shi, Er-yong Zhang

**Affiliations:** 1 Department of Cardiovascular Surgery, West China Hospital, Sichuan University, Chengdu, People’s Republic of China

**Keywords:** Myxoma, Superior vena cava, Echocardiography

## Abstract

Myxomas are the most common primary cardiac tumors. The cardiac myxomas are mostly diagnosed within the atria, and only a few such tumors are reported to have arisen from atrioventricular valves or pulmonary vessels. The authors here present a case of 59-year-old Chinese woman who was hospitalized for exacerbating symptoms of tricuspid stenosis and right heart failure. Echocardiography revealed a giant right atrial myxoma arising from an extremely rare site, the anterior wall of the superior vena cava. With the aid of transesophageal echocardiography, the surgical resection was performed successfully with the patient achieving complete recovery.

## Background

Primary cardiac tumor represents a rare subgroup of malignancies in humans with an incidence between 0.0017 and 0.29 percent in autopsy series performed in non-selected populations [1,2]. The most prevalent type of the intra-cardiac tumor is myxoma, which is approximately 0.5-1 cases per 10^6^ individuals per year, with apparent preponderance (3:1) of female patients [3,4]. Although the majority of cardiac myxomas (CMs) are histologically benign, due to their strategic location (left or right cardiac chamber) and nature (size, mobility and overall morphology), they may lead to serious consequences for morbidity and mortality of affected patients.

The occurrence of CMs is mainly sporadic (90%) and is commonly observed within the left atrium (60-80%) [1,2,5]. As reported by previous studies, about 15-28% of CMs are diagnosed in the right atrium, 8% in the right and 3-4% in the left ventricle, and a small proportion of CMs are biatrial [1,2,6,7]. There are also less common reports of CMs involving all cardiac chambers and originating from mitral leaflets, aortic valves and pulmonary vessels [7-11]. Here we present an extremely rare case of a giant superior vena cava (SVC)-originated myxoma mimicking tricuspid valve obstruction and right heart failure.

## Case presentation

**Figure 1:**
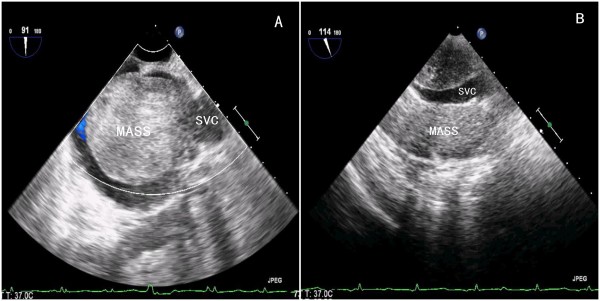
**Transesophageal echocardiography demonstrated a giant mass arising from the SVC. A)** Transesophageal echocardiography revealed a giant mass arising from the anterior wall of the superior vena cava (SVC); **B)** The cavity of the SVC was occupied by the mass.

**Figure 2:**
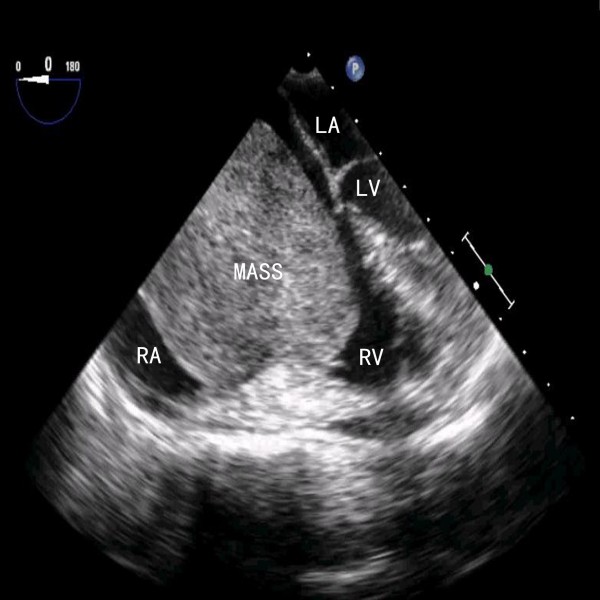
**Transesophageal echocardiography demonstrated an enlarged cavity of the right atrium with the mass prolapsing through the tricuspid orifice into the right ventricle during diastole.** RA, right atrium; RV, right ventricle; LA, left atrium; LV, left ventricle.

A 59-year-old Chinese woman with a 10-year history of exertional dyspnea and palpitations was hospitalized for exacerbating symptoms of right heart failure from one month prior to admission. On physical examination, her pulse rate was 99 beats per minute, and blood pressure was 111/80 mmHg. She had moderate bilateral pitting leg edema, a distended jugular vein and mild dilatation of superficial neck and facial veins. An accentuated second heart sound without tumor plop was heard on auscultation. Electrocardiogram showed sinus rhythm with right bundle branch block. Chest X-ray and abdominal computed tomography were unremarkable. The results of lower extremity ultrasound were negative for deep venous thrombosis. A transesophageal echocardiogram (TEE) demonstrated a mobile and pedunculated giant mass (68 mm × 49 mm) in the enlarged right atrium (70 mm × 53 mm) arising from the anterior wall of the SVC (Figure 1). The mass prolapsed into the right ventricle across the tricuspid valve during systole (Additional file 1: Video 1), resulting in tricuspid orifice obstruction and symptoms of right heart failure (Figure 2).

**Figure 3:**
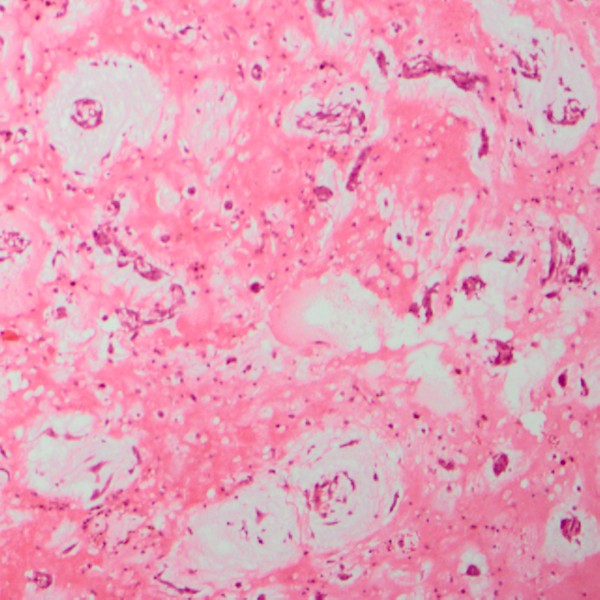
High power field (×200 fold) of a representative section of the myxoma demonstrating a hypocellular benign tumor containing satellite polygonal cells with scant eosinophilic cytoplasm scattered throughout an acid-mucopolysaccharide-rich stroma.

The patient underwent surgical intervention through a median sternotomy and on normothermic cardiac arrest with the aid of cardiopulmonary bypass. To avoid mass fragmentation, care was particularly taken during venous cannulation after pericardiotomy. Briefly, an ultrasound probe was placed transversely on the patient’s right neck, and the short-axis of right internal jugular vein was clearly visualized. A venous cannula (15 Fr) was then inserted into the right internal jugular vein using the Seldinger technique. With the guidance of TEE, the tip of cannula was properly positioned in the distal segment of SVC. Similarly, TEE was performed to assess the location of the myxoma prior to the cannulation of inferior vena cava, keeping the cannulation site as far as possible to prevent a potential mass fragmentation. After opening the right atrium, a grey-green translucent mass in the atrial chamber with a pedicle attached to the anterior wall of the SVC was identified and was then completely excised, no endocardial adhesions in right atrium or ventricle was observed. A glutaraldehyde preserved autologous pericardium was used for patch reconstruction of the anterior wall of the SVC. The tricuspid valve appeared structurally normal, and tricuspid regurgitation was trivial. Histopathologic examination demonstrated a hypocellular benign tumor containing satellite polygonal cells with scant eosinophilic cytoplasm scattered throughout an acid-mucopolysaccharide-rich stroma (Figure 3). Postoperatively, transthoracic echocardiography (TTE) showed normal biventricular function without any residual myxoma and significant tricuspid regurgitation, and the patient had an uneventful recovery and was discharged home at 7 days after surgery.

## Discussion

Myxomas are the most common primary cardiac tumors in adults, constituting up to 50-85% of all benign lesions [1,2,8,12]. As reported by literatures, CMs are more common in women and occur much more frequently between the third and sixth decades of life [1-4]. It is believed that embryonic residues after the in-utero septation of the heart are those that give rise to the myxoma [13]. Thus, CMs may originate from anywhere within the cardiac chambers. Actually, majority of CMs occur in the atria with only 3-10% identified in either the left or right ventricle. The left atrium is the most common site with greater than 75% of all myxomas arising from here, and the right-sided location accounts for only 20% of cases [1,8]. Only a few such tumors are reported to have arisen from atrio-ventricular valves, pulmonary vessels and inferior vena cava [7-11,14,15]. Although there are several literatures reporting CMs-related involvement of SVC, these intra-cardiac neoplasms are exclusively originated from right atrium [16,17]. The present case is of interest because of the rarity of the tumor itself, which had a pedicle from an extremely rare site SVC, occupied almost the entire right atrial cavity, prolapsed into the right ventricle across the tricuspid valve during systole, and caused symptoms resembling tricuspid stenosis and right heart failure. This rare origin has only been reported previously by Teixido et al. [18], however, no signs of tricuspid obstruction and right heart failure were observed.

Besides nonspecific constitutional symptoms such as fevers, arthralgia and erythematous rash, patients with CMs generally present with hemodynamic derangement due to intra-cardiac obstruction and signs of systemic or pulmonary embolization caused by mass fragmentation [1,5,8]. Atrioventricular valve obstruction with corresponding-sided heart failure, as demonstrated in our case, is the major cause of acute presenting symptoms of CMs that facilitate their initial diagnosis. As patients with CMs may demonstrate a wide spectrum of cardiac and non-cardiac symptoms, early and accurate diagnosis is ultimately important in the clinical management of these patients. The echocardiography, both the transthoracic and transesophageal approach, is able to determine the location, size, shape, mobility and attachment of CMs. In the present case, TEE not only characterizes the tumor more clearly than TTE, it also plays a pivotal role in determining cannulation strategy during surgical treatment.

Currently, the treatment of choice for effective therapy of CMs is surgical resection, which is curative. Cardiac surgery should be performed as soon as possible in order to prevent major adverse complications associated with the tumor. As reported by others, the perioperative mortality is low and the long-term prognosis is satisfactory [19,20]. In the present case, due to the size of the mass, we preferred to excise the myxoma during cardiac arrest with the aid of cardiopulmonary bypass. However, it is challenging for us to cannulate the SVC because of the particular location of the mass. Under the guidance of TEE, we managed to drain the SVC blood through the cannulation of the right internal jugular vein and then excise the myxoma completely. Another concern in the surgical treatment of the CMs is the postoperative recurrence. Recurrence after surgical resection of primary lesions has been observed in 1-4% of sporadic and 12-22% of familial cases [1,2,7]. The possible explanations for the CMs recurrence are familial predisposition, unrecognized multifocal origin of primary lesion, incomplete resection or intraoperative dissemination of tumor cells [2,8,12,19]. Therefore, considering the rare origin of this myxoma and the experience learned from Teixido’s report [18], we decided to excise the myxoma and its attachment site (a part of the anterior wall of the SVC) completely, trying to minimize the risk of intra-cardiac recurrence.

## Conclusions

Symptomatic tricuspid obstruction and right heart failure due to intra-cardiac myxoma originating from SVC are extremely rare. With the aid of intraoperative TEE, the surgical removal could be performed successfully and the risk of postoperative recurrence might be minimized.

## Consent

A written informed consent was obtained from the patient’s legal representative for the publication of this case report and any accompanying images.

## Abbreviations

CMs: Cardiac myxomas; SVC: Superior vena cava; TEE: Transesophageal echocardiogram; TTE: Transthoracic echocardiography.

## Competing interests

The authors declare that they have no competing interests.

## Authors’ contributions

ZX and JH conceived, designed, and drafted the manuscript, and performed the surgery. EZ was the chief physician in charge of the patient’s care, and contributed significantly to the manuscript drafting and revision. DZ collected the imaging data. YS involved in the drafting of manuscript. All authors read and approved the final manuscript.

## Supplementary Material

Additional file 1 **The following supplementary material is available online: movie clips for Figures **1** and **2**.** Click here for file
